# The relationship between atherogenic index of plasma and erectile dysfunction: a cross-sectional observational study

**DOI:** 10.1007/s00345-026-06308-1

**Published:** 2026-04-01

**Authors:** Ali Riza Turkoglu, Yasemin Ustundag, Akif Koç, Murat Ozturk, Anil Erkan, Atilla Satir, Oğuzhan Akpinar, Muhammet Guzelsoy, Abdullah Gul, Soner Coban

**Affiliations:** 1 Department of Urology, University of Health Sciences, Bursa School of Medicine, Bursa Yuksek Ihtisas Training and Research Hospital, Bursa, Turkey; 2 Department of Clinical Biochemistry, University of Health Sciences, Bursa Yuksek Ihtisas Training and Research Hospital, Bursa, Turkey; 3 Department of Urology, Bursa Yuksek Ihtisas Training and Research Hospital, Bursa, Turkey

**Keywords:** Adult men, Atherogenic index of plasma, Erectile dysfunction, Testosterone, Lipid profile

## Abstract

**Purpose:**

The aim of this study was to investigate whether lipid profiles and atherogenic indices differ between patients with erectile dysfunction (ED) and men without ED.

**Methods:**

This cross-sectional observational study included 142 patients diagnosed with ED and 54 men without ED men serving as the men without ED group. The presence and severity of ED were assessed using the International Index of Erectile Function questionnaire (IIEF-5). The Atherogenic Index of Plasma (AIP) was calculated as the logarithm of the ratio of plasma triglycerides (TG) to high-density lipoprotein cholesterol (HDL-C).

**Results:**

No significant differences were observed between men with and without erectile dysfunction regarding lipid parameters or atherogenic indices, including AIP. The overall comparison of IIEF-5 scores across AIP categories was not statistically significant, although a modest difference between the lowest and highest AIP groups emerged in an exploratory, unadjusted subgroup analysis. Testosterone levels showed weak but statistically significant inverse correlations with AIP and the TyG index.

**Conclusion:**

In this cohort, atherogenic indices, including AIP, were not independently associated with the presence of erectile dysfunction. Exploratory analyses suggest that higher AIP values may reflect an unfavorable metabolic profile rather than a direct marker of ED severity. These findings should be interpreted cautiously and considered hypothesis-generating.

## Introduction

Erectile dysfunction (ED) is defined as the inability to achieve or maintain an erection sufficient for satisfactory sexual intercourse. It is a common condition worldwide, and its prevalence increases with age [1]. ED shares several risk factors associated with coronary artery disease [2, 3].

Penile erection is maintained by adequate blood flow to the corpus cavernosum. Atherosclerosis is a systemic disease characterized by the accumulation of cholesterol-rich plaques within arterial walls, which may diminish or obstruct blood flow. According to the arterial size hypothesis, penile arteries are affected earlier than larger arteries (such as coronary or carotid arteries), leading to symptoms of ED before clinically apparent cardiovascular disease [4]. Symptoms typically manifest when arterial lumen narrowing reaches approximately 50% [5]. In addition, atherosclerosis induces oxidative stress and endothelial dysfunction, reducing nitric oxide production and release—an essential mediator of normal erectile physiology [6, 7].

Dyslipidemia, diabetes mellitus, and inflammation are among the established risk factors contributing to the development of atherosclerosis. Currently, several biochemical parameters are used to improve prognostic accuracy in atherosclerotic disease, including the Atherogenic Index of Plasma (AIP), logarithmic triglyceride/high-density lipoprotein cholesterol ratio (Log TG/HDL-C), non-HDL cholesterol (non-HDL-C), and the atherogenic coefficient (non-HDL-C/HDL-C) [8, 9]. AIP has been shown to reflect the concentration of atherogenic low-density lipoprotein cholesterol (LDL-C) particles and increases significantly with elevated atherogenic risk [10]. Due to their smaller size, small dense LDL-C particles can more easily penetrate the arterial wall compared to conventional LDL-C particles and are more susceptible to oxidative modification, forming oxidized LDL-C. These oxidized lipoproteins play a crucial role in the development of atherosclerotic lesions and endothelial dysfunction [11].

Although ED arises from multifactorial etiologies, the predominant underlying mechanism is often endothelial dysfunction associated with decreased nitric oxide synthesis and bioavailability, leading to the progression of atherosclerosis [12].

The aim of this study was to investigate whether lipid profiles and atherogenic indices differ between patients with ED and men without ED men.

## Materials and methods

Adult male patients who presented to the Urology Clinic of the Bursa Training and Research Hospital in 2018 were considered potential participants for this cross-sectional observational study. Ethical approval was obtained from the Regional Ethics Committee (2011-KAEK-25 2016/22-01). The study was conducted in accordance with the Declaration of Helsinki.

Exclusion criteria included untreated or insufficiently treated endocrine disorders, penile fracture or Peyronie’s disease, neurological disorders, a history of malignancy, pelvic trauma, psychiatric disease, acute or chronic urinary tract disease, renal failure, hepatobiliary disease, and erectile dysfunction secondary to these conditions. Patients using medications known to affect ED (e.g., diuretics, beta blockers) were also excluded.

For the purposes of this study, exclusion criteria were operationally defined as follows: untreated or insufficiently treated endocrine disorders referred to documented endocrine conditions known to affect sexual function, such as hypogonadism, uncontrolled thyroid disease, or hyperprolactinemia, that were either untreated or not adequately controlled according to medical records. Chronic urinary tract disease included active or recurrent conditions such as chronic prostatitis, interstitial cystitis, neurogenic bladder, or a history of major lower urinary tract surgery. Psychiatric disease referred to clinically diagnosed psychiatric disorders requiring ongoing pharmacological treatment or with documented impact on sexual function.

Erectile function was assessed using the five-item International Index of Erectile Function (IIEF-5). The men without ED group consisted of men without ED individuals with IIEF-5 scores ranging from 22 to 25. Smoking status and body mass index (BMI) were recorded. BMI was calculated as weight in kilograms divided by height in meters squared (kg/m²).

Hemogram parameters were analyzed using LH 780 analyzers (Beckman Coulter Inc., Fullerton, CA). Serum glucose, total cholesterol (TC), triglycerides (TG), HDL-C, and creatinine were measured using commercial assay kits on the Olympus AU 2700 system (Beckman Coulter Inc., Fullerton, CA, USA). C-reactive protein (CRP) levels were determined using the Siemens BNII nephelometric method (Siemens Healthcare Diagnostics, Tarrytown, NY, USA), and testosterone levels were obtained via the Siemens Advia Centaur XP immunoassay system (Siemens Healthcare Diagnostics, Tarrytown, NY, USA) from the hospital database.

The Atherogenic Index of Plasma (AIP) was calculated using the logarithm of the ratio of plasma TG to HDL-C concentration. The Triglyceride-Glucose Index (TyG) was calculated using the formula TyG = ln (fasting TG (mg/dL) × fasting glucose (mg/dL)/2), as described by Lee et al. [22]. Participants were allocated into two primary categories: the men without ED group and the ED group. All participants were sexually active men with a regular sexual life for at least six months. Informed written consent was obtained from all subjects following detailed study explanation.

### Statistical analysis

All statistical analyses were performed using SPSS version 20.0 (SPSS for Windows 10.0, Chicago, IL, USA). The normality of data distribution was assessed using the Kolmogorov–Smirnov test. Continuous variables were expressed as mean ± standard deviation (SD) or median (interquartile range), as appropriate. Statistical differences between groups were analyzed using the Student’s t-test or Mann–Whitney U test, depending on data distribution. Correlations between variables were evaluated using Spearman’s correlation analysis. Subgroup analyses were exploratory in nature, and no adjustment for multiple comparisons was applied. A p-value < 0.05 was considered statistically significant.

## Results

When participants were categorized into two groups—the men without ED group (*n* = 54) and patients with ED (*n* = 142)—none of the evaluated parameters differed significantly between the groups (Table 1).

**Table 1:** Sociodemographic, characteristics and laboratory data of the studied subjects

Number of patients	Men without ED	Men with ED	*p*
	54	142	
Age (years)	48.6 ± 9.7	49.3 ± 9.8	0.677
BMI (kg/m^2^)	27.0 ± 3.4	26.9 ± 3.5	0.763
IIEF score	25(IQR: 2)	13(7)	**< 0.001**
Fasting glucose (mg/dl)	102(19)	100(16)	0.733
Creatinine (mg/dl)	0.97 ± 0.14	0.96 ± 0.14	0.719
AST	21(5)	21(8)	0.445
Total cholesterol (mg/dl)	217 ± 49	207 ± 45	0.169
LDL (mg/dl)	135(66)	127(52)	0.421
Triglyceride (mg/dl)	135(87)	151(114)	0.771
HDL (mg/dl)	46(12)	43(11)	0.074
TC/HDL	4.8 ± 1.3	4.9 ± 1.2	0.727
LDL/HDL	2.8 ± 1.2	2.9 ± 1.2	0.637
Non HDL cholesterol (mg/dl)	171 ± 48	163 ± 43	0.278
Atherogenic coefficient	3.5(1.6)	3.8(1.4)	0.604
Tyg (mg/dl)	4.7 ± 0.3	4.8 ± 0.3	0.737
AIP	0.13 ± 0.24	0.17 ± 0.27	0.264
CRP (mg/l)	3.5 ± 1.4	3.5 ± 1.4	0.790
WBC	7.8(2.4)	7.2(7.2)	0.171
MPV	9.2(1.5)	8.7(1.4)	0.307
RDW	13.2(1.0)	13.1(0.9)	0.234
Testosterone (ng/dl)	399 ± 110	414 ± 156	0.517

*ED* erectile dysfunction *BMI* body mass index, *IIEF* International Index of Erectile Function, *AST* aspartat aminotransferaz, *LDL* low density lipoprotein, *HDL* high density lipoprotein, *TC* total cholesterol, *TyG* tryglyceride glucose index, *AIP* atherogenic index plasma, *CRP* C-reactive protein, *WBC* White blood cell; *MPV* mean platelet volume; *RDW* red cell distribution width

Bold values indicate statistical significance (p<0.05).

Participants were further stratified into five groups according to their IIEF-5 scores: Group 1 (*n* = 23, scores 5–7), Group 2 (*n* = 33, scores 8–11), Group 3 (*n* = 54, scores 12–16), Group 4 (*n* = 32, scores 17–21), and Group 5, the men without ED group without ED (*n* = 54, scores > 21) (Table 2).

**Table 2:** Data of groups according to IIEF scores

	Group 1 severe (5–7skor)	Group 2 moderate (8–11skor)	Group 3 mild to moderate (12–16skor)	Group 4 mild erectile (17–21skor)	Group 5 men without ED (22–25skor)	*p*
Number of patients	23	33	54	32	54	
IIEF: score	6.0(5–7)	10 (8–11)	13(12–16)	19(17–21)	25(22–25)	**< 0.001**
BMI (kg/m^2^)	27.8 ± 3.0	26.8 ± 3.6	26.4 ± 3.7	27.0 ± 3.4	27.0 ± 3.4	0.641
Age (years)	53.2 ± 9.8	49.7 ± 10.5	49.5 ± 9.4	45.5 ± 8.6	48.0 ± 9.6	0.085
Smoking (%)	46%	52%	54%	29%	43%	
Fasting glucose (mg/dl)	103(16)	99(17)	99(16)	101(17)	102(12)	0.971
Creatinine (mg/dl)	0.98 ± 0.10	0.95 ± 0.16	0.96 ± 0.15	0.96 ± 0.16	0.97 ± 0.14	0.967
Total cholesterol(mg/dl)	197 ± 47	201 ± 43	211 ± 45	211 ± 45	217 ± 49	0.295
LDL (mg/dl)	121 ± 42	124 ± 36	138 ± 42	138 ± 43	130 ± 54	0.312
Triglyceride (mg/dl)	164 ± 83	161 ± 84	188 ± 104	153 ± 86	174 ± 100	0.865
HDL (mg/dl)	43 ± 10	43 ± 11	43 ± 9	46 ± 8	46 ± 9	0.423
TC/HDL	4.7 ± 1.2	4.7 ± 1.0	5.0 ± 1.1	4.9 ± 1.5	4.8 ± 1.2	0.812
LDL/HDL	2.7 ± 1.0	2.8 ± 0.8	3.1 ± 1.1	2.8 ± 1.2	2.8 ± 1.1	0.467
Non HDL cholesterol	152 ± 45	159 ± 38	169 ± 43	168 ± 47	171 ± 48	0.380
Atherogenic coefficient	3.7 ± 1.1	3.7 ± 1.0	4.0 ± 1.1	3.9 ± 1.5	3.8 ± 1.2	0.870
Tyg index (mg/dl)	4.7 ± 0.3	4.7 ± 0.3	4.8 ± 0.3	4.8 ± 0.3	4.7 ± 0.3	0.636
AIP	0.16 ± 0.33	0.16 ± 0.27	0.21 ± 0.25	0.11 ± 0.27	0.15 ± 0.25	0.668
CRP (mg/l)	3.1(0.3)	3.3(0.2)	3.2(3.0)	3.1(0.2)	3.2(0.1)	0.248
WBC	8.2 ± 1.9	7.9 ± 2.4	7.6 ± 1.9	7.0 ± 1.5	8.0 ± 1.9	0.311
MPV	8.5 ± 9	9.1 ± 12	9.0 ± 11	9.0 ± 12	9.2 ± 10	0.119
RDW	13.3 ± 1.1	13.3 ± 1.1	13.1 ± 1.1	13.0 ± 0.3	12.9 ± 0.2	0.944
Testosterone (ng/dl)	420 ± 196	418 ± 142	405 ± 154	426 ± 142	400 ± 107	0.814

*IIEF* International Index of Erectile Function, *BMI* body mass index, *LDL* low density lipoprotein, *HDL* high density lipoprotein, *TC* total cholesterol, *TyG* tryglyceride glucose index, *AIP* atherogenic index plasma, *CRP* C-reactive protein, *WBC* White blood cell; *MPV* mean platelet volume; *RDW* red cell distribution width

Bold values indicate statistical significance (p<0.05).

**Fig. 1:**
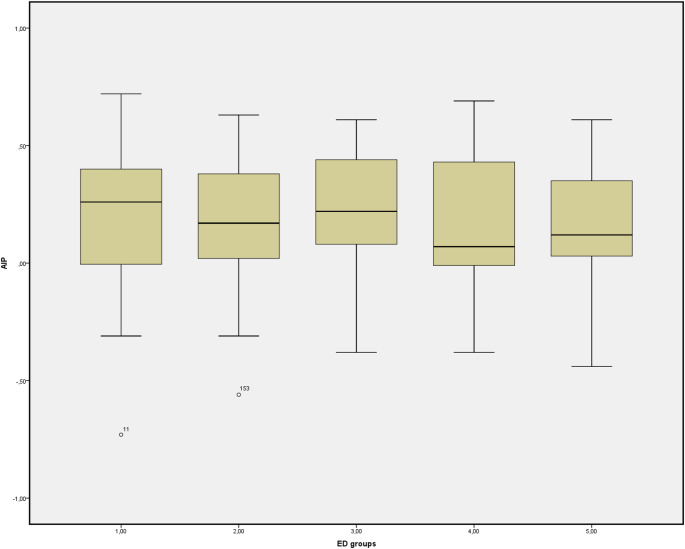
The atherogenic index of the patients according to severity of ED and men without ED groups

Based on ED severity classifications determined by IIEF-5 scores, BMI was comparable among all ED subgroups and the men without ED group (*p* = 0.641) (Table 2). The mean AIP value was 0.16 ± 0.33 in patients with severe ED and 0.15 ± 0.25 in the men without ED group, with no significant differences across ED severity groups (Fig. 1).

**Fig. 2:**
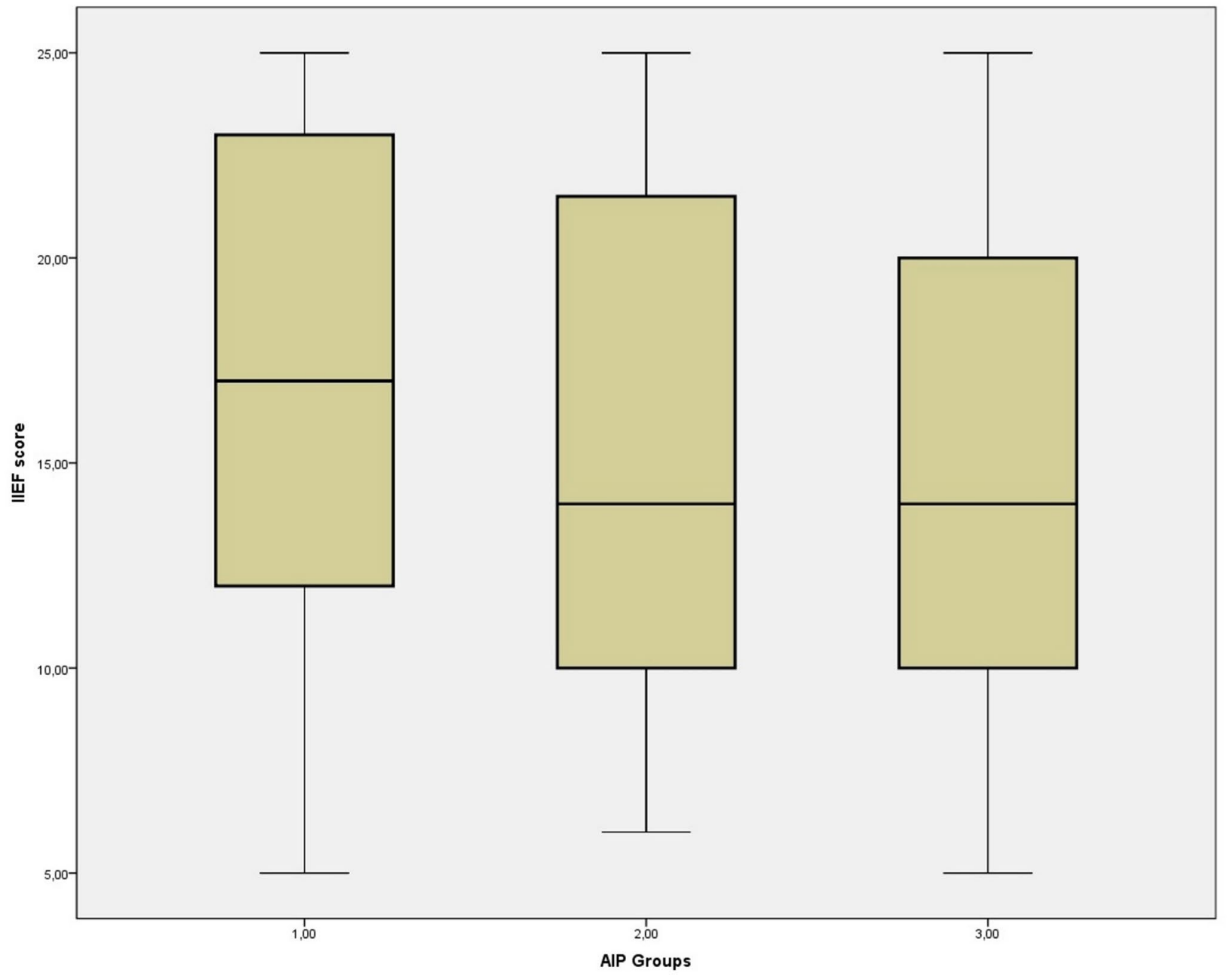
Exploratory comparison of IIEF-5 scores across AIP categories

Participants were also categorized according to AIP level, and their IIEF-5 scores were compared. Group 1 included patients with low atherosclerotic risk (AIP < 0.10, *n* = 77), Group 2 included those with borderline risk (AIP 0.10–0.24, *n* = 33), and Group 3 consisted of individuals with high atherosclerotic risk (AIP ≥ 0.24, *n* = 86). The median IIEF-5 scores were 17 (IQR: 11), 14 (IQR: 12), and 14 (IQR: 10) for Groups 1, 2, and 3, respectively (*p* = 0.089). Although the overall comparison across AIP categories was not statistically significant, a modest difference between the lowest and highest AIP groups was observed in an exploratory, unadjusted pairwise comparison (Fig. 2).

A statistically significant negative correlation was observed between testosterone and both AIP (*r* = − 0.191, *p* = 0.007) and the TyG index (*r* = − 0.230, *p* < 0.001). No significant correlation was found between IIEF-5 scores and any of the evaluated clinical or biochemical parameters (Table 3).

**Table 3:** Correlation between testosterone, triglyceride–glucose index, and AIP

	Testosterone	TyG
Age		
r	0.015	0.061
p	0.835	0.395
BMI		
r	− 0.034	− 0.030
p	0.636	0.675
AIP		
r	− 0.191	0.864
p	0.007*	0.000*
CRP		
r	− 0.70	0.023
p	0.333	0.745
Fasting glucose		
r	− 0.192	0.306
p	0.007*	0.00*
LDL/HDL		
r	0.056	− 0.125
p	0.434	0.082
TC/HDL		
r	− 0.076	0.237
p	0.293	0.00*
Non HDL/HDL		
r	− 0.076	0.455
p	0.293	0.00*
TyG		
r	− 0.230	
p	0.00*	

*BMI* body mass index, *AIP* atherogenic index plasma, *CRP* C-reactive protein, *LDL* low density lipoprotein, *HDL* high density lipoprotein, *TC* total cholesterol, *TyG* tryglyceride glucose index

*p value < 0.05 considered statistically significant

## Discussion

The aim of this study was to investigate whether lipid profiles and atherogenic indices differ between patients with ED and their men without ED counterparts. In this study, no significant differences were observed in lipid profiles or atherogenic indices between men with ED and those without erectile dysfunction. Although exploratory subgroup analyses suggested modest differences in erectile function across AIP categories, these findings were not supported by correlation analyses and should be interpreted with caution.

In our study population, no association was found between IIEF-5 scores and atherogenic risk indices. Consistent with our findings, Lojanapiwat et al. reported that TC, TG, LDL-C, and testosterone levels did not differ between patients with and without ED; however, HDL-C levels were significantly lower in the ED group [5]. Conversely, Eaton et al. demonstrated that men with ED had a higher TC/HDL-C ratio compared with those with normal erectile function, suggesting that the TC/HDL-C ratio may serve as a predictive risk marker for ED [13, 14].

When participants were stratified according to AIP categories, individuals in the highest AIP group demonstrated modestly lower IIEF-5 scores compared with those in the lowest AIP group. However, this finding emerged from an exploratory, unadjusted subgroup comparison and was not supported by correlation analyses using continuous AIP values. Moreover, no significant differences in AIP were observed when participants were stratified according to ED severity, including comparisons between severe ED and men without ED group. Taken together, these results suggest that the observed subgroup difference should not be interpreted as evidence of a direct or linear association between atherogenic burden and ED severity.

Importantly, the absolute difference in IIEF-5 scores between AIP categories was small, with overlapping interquartile ranges, raising questions regarding its clinical relevance. Therefore, while higher AIP values may reflect a less favorable metabolic profile, the present findings do not support the use of AIP as a reliable marker of ED severity in routine clinical practice.

Atherosclerosis progresses silently before clinical cardiovascular symptoms manifest [15]. During this subclinical period, plaque burden may first affect smaller arteries, potentially contributing to the early presentation of ED symptoms. AIP has been proposed as a marker reflecting adverse cardiometabolic risk, particularly in population-based cardiovascular studies. However, emerging evidence suggests that the corporal and trabecular vascular networks may not behave identically to systemic cardiovascular structures. Ponholzer et al. demonstrated that penile arterial lesions occur infrequently among patients with coronary and peripheral atherosclerosis, observed in only 13% of cases [16].

Sambel et al. reported higher values of several metabolic and atherogenic indices, including AIP and the TyG index, in men with erectile dysfunction, and identified these parameters as independent predictors within their analytical model. However, differences in study design, population characteristics, and statistical adjustment may account for the stronger associations reported in that study compared with the findings of the present analysis. They also suggested that hyperlipidemia may contribute to ED by affecting the vascular endothelium and smooth muscle cells of the penis, that LDL is an important biomarker for ED, and that it may play a causal role in impaired relaxation responses of the corpus cavernosum [17].

Guo et al. investigated the association of AIP, AC, CRI-I, and CRI-II with ED by analyzing data from 1,806 individuals in the 2001–2004 National Health and Nutrition Examination Survey (NHANES). In contrast to our findings, Guo et al. reported an association between higher AIP levels and ED prevalence in a large population-based cohort after multivariable adjustment [18].

Atherogenesis is increasingly recognized as a multifactorial and time-dependent process involving chronic low-grade inflammation, insulin resistance, hormonal imbalance, and cumulative metabolic exposure rather than isolated lipid abnormalities [6, 19]. Endothelial dysfunction represents a central mechanism linking these metabolic disturbances to vascular disease, affecting both systemic and penile vasculature through impaired nitric oxide bioavailability and altered vascular reactivity [4, 6]. In this context, lipid-derived composite indices such as the atherogenic index of plasma (AIP) should be interpreted as indirect markers of an adverse cardiometabolic milieu rather than direct surrogates of structural atherosclerotic burden [10]. This distinction is particularly relevant in erectile dysfunction, where endothelial dysfunction and microvascular impairment may precede or occur independently of overt macrovascular atherosclerosis, consistent with the arterial size hypothesis and emerging evidence on microvascular disease in ED [4, 16].

The lack of significant differences in AIP and conventional lipid parameters between men with and without erectile dysfunction in our study further supports a cautious interpretation of AIP-related findings. While several large population-based studies have reported associations between elevated AIP values and erectile dysfunction prevalence after comprehensive multivariable adjustment, these associations appear to be context-dependent and are not consistently observed across smaller, clinically characterized cohorts [17, 18, 20]. Differences in study design, population characteristics, outcome definitions, and analytical approaches likely contribute to this heterogeneity. Accordingly, the present findings should be regarded as hypothesis-generating rather than confirmatory and underscore the need for larger, prospective studies incorporating detailed metabolic, hormonal, and vascular assessments to better clarify the clinical relevance of atherogenic indices in erectile dysfunction [17, 20].

Our findings are consistent with previous studies reporting no significant association between serum total testosterone levels and IIEF scores [21, 22]. Testosterone, the primary male sex hormone produced predominantly by the testes, is known to influence both cardiovascular and erectile function [23]. Endothelial cells serve as a major target for the beneficial vascular effects of androgen hormones [6]. Furthermore, recent evidence has shown that testosterone exerts protective effects against atherogenesis [24, 25]. Normal plasma testosterone concentrations contribute to a favorable lipid profile by lowering total cholesterol and LDL-C levels. A significant inverse correlation between testosterone and AIP, as well as between testosterone and glucose levels, has previously been demonstrated. Notably, AIP was not evaluated in the study by Dunajska et al., and therefore their findings should be interpreted in the context of general metabolic associations rather than atherogenic indices [26].

In addition, we found a significant inverse correlation between testosterone and the TyG index, a simple surrogate marker of insulin resistance [27]. The clinical consequences of insulin resistance commonly include dyslipidemia, characterized by elevated plasma triglycerides, reduced HDL-C levels, the presence of small dense LDL-C particles, and abnormal vascular behavior [28]. Moreover, recent studies have demonstrated that the TyG index correlates with angiographically confirmed coronary artery disease and may reflect underlying cardiovascular pathology [29, 30].

This study has several limitations. First, the cross-sectional design precludes assessment of temporality and causal relationships. Second, the relatively small sample size limits statistical power, particularly for subgroup analyses. Third, the absence of multivariable adjustment restricts the ability to account for potential confounding factors such as age, metabolic comorbidities, lifestyle variables, and medication use. Finally, multiple exploratory subgroup analyses were performed without adjustment for multiple comparisons, and these findings should therefore be interpreted with caution. Therefore, routine lipid evaluation should be considered within the broader context of comprehensive cardiometabolic risk assessment rather than as a specific tool for ED prediction. Given the largely negative primary findings, multivariable modeling was not pursued, as it was unlikely to yield clinically meaningful independent associations in this cohort.

In conclusion, the present findings do not support the use of the atherogenic index of plasma as a standalone biomarker for ED. While AIP may reflect an adverse metabolic milieu, its clinical utility in the evaluation of ED appears limited in the absence of comprehensive multivariable risk assessment. Larger, well-designed prospective studies with comprehensive multivariable adjustment are required to clarify the clinical relevance of atherogenic indices in ED.

## Data Availability

Data is provided within the manuscript. Data anonymised at source prior to collection. For raw data please contact author.
